# Barley (*Hordeum vulgare*) circadian clock genes can respond rapidly to temperature in an *EARLY FLOWERING 3*-dependent manner

**DOI:** 10.1093/jxb/erw317

**Published:** 2016-08-31

**Authors:** Brett Ford, Weiwei Deng, Jenni Clausen, Sandra Oliver, Scott Boden, Megan Hemming, Ben Trevaskis

**Affiliations:** CSIRO Agriculture, GPO Box 1600, Canberra, ACT 2601, Australia

**Keywords:** Barley, circadian clock, *elf3,* flowering, temperature.

## Abstract

An increase in global temperatures will impact future crop yields. In the cereal crops wheat and barley, high temperatures accelerate reproductive development, reducing the number of grains per plant and final grain yield. Despite this relationship between temperature and cereal yield, it is not clear what genes and molecular pathways mediate the developmental response to increased temperatures. The plant circadian clock can respond to changes in temperature and is important for photoperiod-dependent flowering, and so is a potential mechanism controlling temperature responses in cereal crops. This study examines the relationship between temperature, the circadian clock, and the expression of flowering-time genes in barley (*Hordeum vulgare*), a crop model for temperate cereals. Transcript levels of barley core circadian clock genes were assayed over a range of temperatures. Transcript levels of core clock genes *CCA1*, *GI*, *PRR59*, *PRR73, PRR95*, and *LUX* are increased at higher temperatures. *CCA1* and *PRR73* respond rapidly to a decrease in temperature whereas *GI* and *PRR59* respond rapidly to an increase in temperature. The response of *GI* and the *PRR* genes to changes in temperature is lost in the *elf3* mutant indicating that their response to temperature may be dependent on a functional *ELF3* gene.

## Highlight

In barley, *GIGANTEA* and the *PSEUDO RESPONSE REGULATOR* clock genes respond rapidly to temperature in an *EARLY FLOWERING 3*-dependent manner.

## Introduction

The flowering time and yield of temperate cereals is strongly influenced by temperature (Fischer, 1985; Hemming *et al.*, 2012). In barley (*Hordeum vulgare*), elevated ambient temperatures accelerate vegetative growth and reduce biomass (Hemming *et al.*, 2012). Reproductive development is accelerated at higher temperatures in long days, resulting in shorter spikes, fewer total florets, and an overall decrease in grain yield (Fischer, 1985; Hemming *et al.*, 2012; Rawson and Richards, 1993). By contrast, in short days, reproductive development is inhibited at higher temperatures; indicating an interaction between photoperiod and temperature in the control of reproductive development in barley (Hemming *et al.*, 2012). Despite predicted global climate change and the negative impact of higher temperatures on cereal grain yield, little is known about the genes and molecular pathways controlling temperature-dependent growth and development in cereal crops.

The plant circadian clock comprises a network of interlocking gene loops that generate and maintain rhythmic gene expression over an approximately 24h period and, in turn, control many rhythmic biological processes (Harmer, 2009). The circadian clock also tracks changing day length to regulate photoperiod-dependent flowering (Imaizumi, 2010). An intrinsic property of the plant circadian clock is the ability to perceive and respond to temperature cues. Firstly, in a process termed ‘temperature entrainment’, the daily rhythmic expression of clock genes can be synchronized to daily temperature changes (Salome and McClung, 2005). Secondly, the plant circadian clock maintains rhythmic gene expression across a wide range of temperatures in a process termed ‘temperature compensation’ (Edwards *et al.*, 2005; Gould *et al.*, 2006). This ability of the plant circadian clock to perceive and integrate temperature cues and its role in photoperiod-dependent flowering make it a strong candidate for having a role in the control of temperature-dependent flowering in the cereals.

The plant circadian clock is best described in *Arabidopsis thaliana*. The key components of the plant circadian clock are the *MYELOBLASTOSIS* (*MYB*)-related genes *CIRCADIAN CLOCK ASSOCIATED 1* (*CCA1*) and *LATE ELONGATED HYPOCOTYL* (*LHY*), the *PSEUDO RESPONSE REGULATORS* (*PRRs*), *PRR5*, *PRR7*, *PRR9*, *TIMING OF CAB EXPRESSION 1* (*TOC1* / *PRR1*), and the members of the evening complex; *LUX ARRHYTHMO* (*LUX*) / *PHYTOCLOCK1* (*PCL1*), *EARLY FLOWERING 3* (*ELF3*), and *ELF4* (Schaffer *et al.*, 1998; Somers *et al.*, 1998; Wang and Tobin, 1998; Covington *et al.*, 2001; Doyle *et al.*, 2002; Hazen *et al.*, 2005; Nakamichi *et al.*, 2005a, b). Mathematical modelling of the plant circadian clock suggests that rhythmic gene expression is maintained by a three loop repressor model (Pokhilko *et al.*, 2012). The morning-expressed *CCA1* and *LHY* are repressed sequentially throughout the day by the protein products of *PRR9*, *PRR7*, and *TOC1* which are, in turn, repressed in the evening by the evening complex (Pokhilko *et al.*, 2012). Finally, the loop is closed with repression of the evening complex genes by CCA1 and LHY in the morning (Pokhilko *et al.*, 2012). In addition, *GIGANTEA* (*GI*) is integral to maintaining circadian rhythms (Fowler *et al.*, 1999; Park *et al.*, 1999). *CCA1*, *LHY*, *TOC1*, *PRR7*, *PRR9, ELF3,* and *GI* have all been shown to have a role in either temperature entrainment or temperature compensation (Salome and McClung, 2005; Gould *et al.*, 2006; Paltiel *et al.*, 2006; Salome *et al.*, 2010; Thines and Harmon, 2010).

A recent study has proposed a model to explain how temperature cues are integrated into the clock; warm temperatures inhibit the function of the evening complex, removing repression of *PRR7*, *PRR9*, *GI,* and *LUX* (Mizuno *et al.*, 2014). The resultant increase in expression of these clock genes may contribute to temperature entrainment, temperature compensation, as well as temperature-dependent growth, development, and flowering (Mizuno *et al.*, 2014). Consistent with this hypothesis, a *prr5/prr7/prr9* triple mutant mimics normal low temperature responses (Nakamichi *et al.*, 2009).

Many equivalent clock genes to those of Arabidopsis and rice (*Oryza sativa*) have been identified in barley (Campoli *et al.*, 2012*b*; Calixto *et al.*, 2015). The similarity of their amino acid sequence, and daily expression patterns, suggest that the function of most of the barley and Arabidopsis clock genes may be conserved (Campoli *et al.*, 2012*b*; Calixto *et al.*, 2015). Some significant differences do exist; in barley, there is no clear homologue of the Arabidopsis evening-expressed *ELF4* gene, although two related *ELF4-like* genes are present (Calixto *et al.*, 2015) and there is only one functionally equivalent morning-expressed gene that is more closely related to *LHY* than *CCA1* (Calixto *et al.*, 2015). In addition, unlike Arabidopsis, the barley circadian clock does not initiate robust rhythmic gene expression until it has been exposed to both a lights-on and a lights-off cue (Deng *et al.*, 2015). It also responds rapidly to different photoperiods, adjusting the period of the clock to match the prevailing photoperiod conditions (Deng *et al.*, 2015). Potentially, the barley circadian clock also responds differently to temperature cues.

Flowering time mutants have recently been identified as mutations of barley clock genes. The barley *PPD-H1* gene is homologous to *PRR3/PRR7* and mediates the acceleration of development in long-days. No similar role exists for *PRR3* or *PRR7* in Arabidopsis (Turner *et al.*, 2005; Campoli *et al.*, 2012*b*). The barley *elf3 (Mat.a8, eam8*) mutant displays disrupted expression of clock genes and accelerated reproductive development (Faure *et al.*, 2012; Zakhrabekova *et al.*, 2012; Boden *et al.*, 2014). The probable *lux* mutants in barley (*eam10*) and diploid wheat (*Triticum monococcum*) (*Eps-3A*) are also early flowering and the *Eps-3A* mutant displays a temperature-dependent variation in spikelet number (Campoli *et al.*, 2013; Gawronski *et al.*, 2014).

Currently, there is little understanding of how temperature cues are integrated into the cereal circadian clock and how this might regulate temperature-dependent growth and development. Given the potential for reduced cereal yields in a warming environment, an in-depth study of temperature and the cereal circadian clock is required. This study examines the expression of core clock genes at high and low ambient temperatures in barley. We show that the barley circadian clock responds to temperature through significant changes in the expression of core clock genes.

## Materials and methods

### Plant material and growth conditions

Plant materials used in this study were the winter-type, photoperiod-sensitive, barley cultivar Sonja (*VRN1, VRN2, PPD-H1*) (Sasani *et al.*, 2009), the barley *elf3* (*Mat.a8*, *eam8*) mutant, and the isogenic spring-type, photoperiod-insensitive, wild-type parent cultivar of the *elf3* mutant, Bonus (*VRN1-1, ΔVRN2, ppd-H1*) (Faure *et al.*, 2012; Zakhrabekova *et al.*, 2012). Barley seeds were sown on a mix of 50% perlite:50% vermiculite in 15ml Falcon tubes and saturated in water supplemented with 1.4g l^-1^ Thiram fungicide (Bayer Crop Science, www.bayercropscience.us) as previously described (Deng *et al.*, 2015). Barley seeds of cultivar Sonja were vernalized in the dark at 4 °C for 28 d.

### Temperature entrainment experiment

**Fig. 1.**
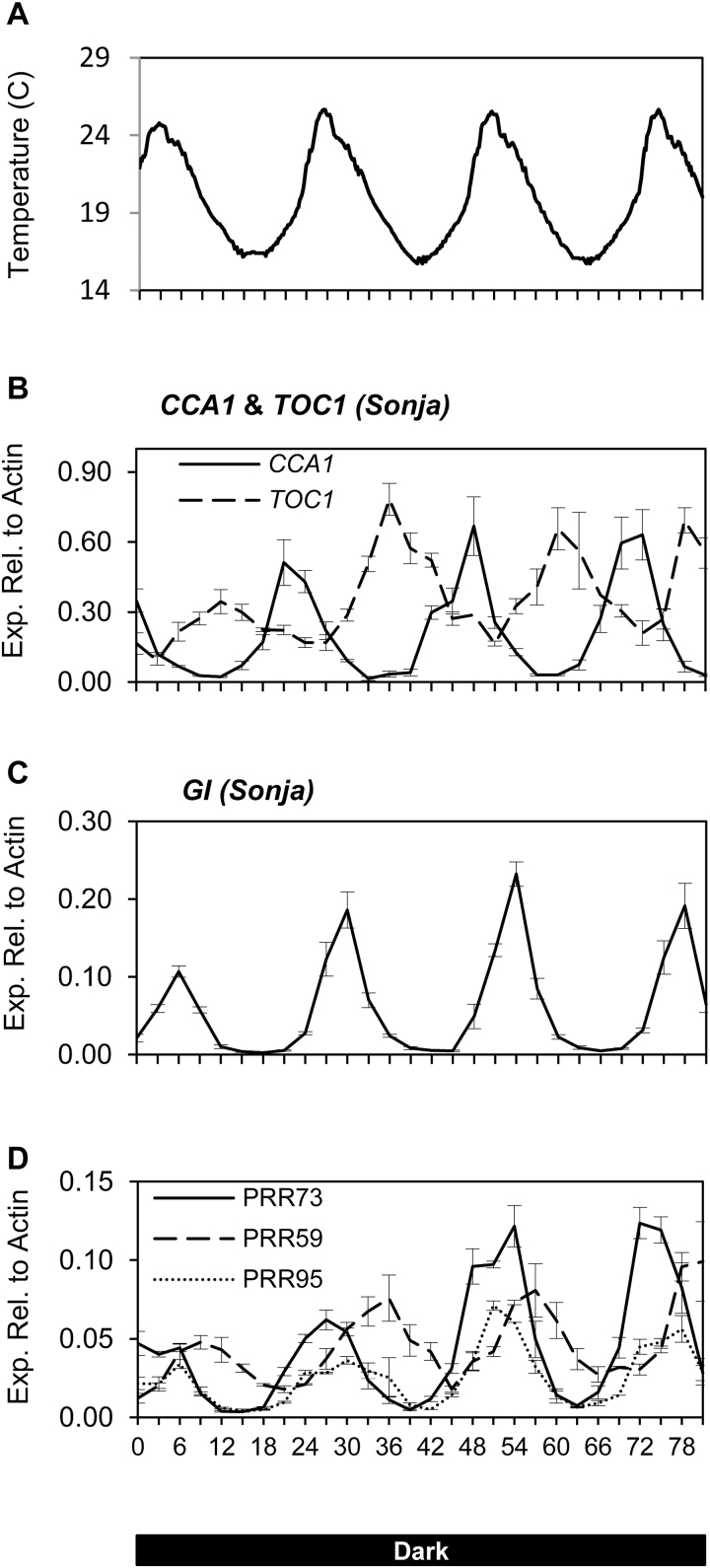
Expression of circadian clock genes in barley cv. Sonja entrained to temperature cycles in constant dark. Relative expression of circadian clock genes in 5-d-old barley seedlings, cv. Sonja, grown in constant dark with a daily temperature range of 16–25.5 °C. (A). Daily temperature range for the duration of the experiment. (B–D). Expression of circadian clock genes. Values are means of three biological replicates ±standard error.

Temperature entrainment experiments were conducted in constant darkness in a temperature-controlled glasshouse with a temperature range of 16–25.5 °C. Temperature was monitored with a data logger with the temperature changing gradually over a 24h period (Fig. 1A). Five days after germination, barley seedlings (whole plant minus the roots) were harvested every 3h for 81h (Sonja) or 24h (Bonus and *elf3*).

### Twelve hour temperature shift from constant conditions

Temperature shift experiments were conducted on barley seedlings germinated and grown for 5 d in darkness at a constant 20 °C, in Ecotherm programmable temperature blocks. The growth temperature was then either increased to 25 °C or decreased to 15 °C for 12h before being returned to 20 °C. Gene expression from the temperature increase or decrease was compared with control seedlings maintained at 20 °C. Data from Sonja control seedlings has previously been presented in Deng *et al.* (2015). Individual barley seedlings were harvested every 3h from the initial temperature increase or decrease for 24h.

### Constant temperature treatments in long days

Long-day experiments were performed in controlled environment cabinets (Conviron CMP6050) set to 16/8h light/dark with a light intensity of 250 µmol m^−2^ s^−1^. Barley seedlings of cv. Sonja were germinated and grown for 5 d at a constant 15, 20, 25 or 30 °C in Ecotherm programmable temperature blocks. For analysis of daily gene expression at a constant 15 °C or 25 °C, individual barley seedlings of cv. Sonja were harvested every 3h for 24h. For analysis of the dosage response to temperature at a constant 15, 20, 25 or 30 °C, individual barley seedlings of cv. Sonja were germinated and grown for 5 d and then harvested at 1, 4, 7, 10, and 16h after lights on.

Bonus and *elf3* seedlings were germinated and grown at a constant 15 °C or 25 °C in Ecotherm programmable temperature blocks, in 16/8h light/dark, for 4 d to allow for entrainment of the barley circadian clock. Constant dark conditions were then imposed just prior to the light period at the start of day 5 by covering the tubes in foil. Seedlings were grown for a further 24h in constant dark and then individual seedlings were harvested every 4h for 24h.

### Six hour temperature increase in long days

Barley seedlings cv. Sonja were germinated and grown for 5 d in long-day conditions 16/8h light/dark at a constant 15 °C in Ecotherm programmable temperature blocks. The temperature was then increased to 25 °C for 6h on day 6 at 1, 7, and 15h, after lights on. Individual barley seedlings were harvested immediately prior to and after the 6h temperature increase.

### Seedling growth experiment

Seedlings of the barley cultivar Sonja were germinated and grown as previously described at a constant 15, 20, 25 or 30 °C in Ecotherm programmable temperature blocks in long days. Seedlings were harvested every day after germination for 3 d for measurement of seedling length.

### Gene expression analyses

To assess gene expression patterns, individual barley seedlings (whole plant minus the roots) were harvested, with seedlings sampled during dark periods harvested in the dark. RNA was extracted using the Spectrum™ Plant Total RNA Kit (Sigma-Aldrich) as per the manufacturer’s instructions or by the method of Chang *et al.* (1993). Total RNA (2 µg) was DNase treated using the On-column DNase I Digestion set (Sigma-Aldrich) or RQ1 DNase (Promega) as per the manufacturers’ instructions. First strand cDNA synthesis primed with Oligo dT(18) was performed using Maxima H Minus Reverse Transcriptase (Thermo-Scientific) as per the manufacturer’s instructions. Quantitative real time reverse transcriptase PCR (qRT-PCR) was performed using the 7900HT Fast Real-Time PCR System (Applied Biosystems), using Platinum Taq DNA polymerase (Life Technologies) and SYBR green. *ACTIN* was used as the reference gene as it is the standard reference gene for barley clock studies and has been previously shown to be stable at different temperatures (Campoli *et al.*, 2012*b*, 2013; Faure *et al.*, 2012; Hemming *et al.*, 2012; Deng *et al.*, 2015). *GAPDH* was also used as a reference gene on a small subset of experiments with results similar to those obtained using *ACTIN* (see Supplementary Fig. S1 at *JXB* online). Relative transcript levels were calculated using the ΔΔCt method allowing for primer amplification efficiencies as previously described by Deng *et al.* (2015). Appropriate no template controls were included and melt curve analysis conducted for all qRT-PCR experiments. qRT-PCR results are the mean values of at least three biological replicates. Sequences of qRT-PCR primers used in this study have previously been described (Deng *et al.*, 2015). Additional primers used in this study are listed in see Supplementary Table S1 at *JXB* online. Barley circadian clock gene sequences (*CCA1*, *PRR59*, *PRR73*, *PRR95*, *TOC1*, and *GI*) have previously been described (Campoli *et al.*, 2012*b*). Sequences of *VRN1*, *VRN2*, *PPD1*, *FT1*, *FT2*, *FT4*, *FT5*, *TFL1*, *FPF1-like1*, *FPF1-like2*, *FPF1-like3*, *ELF3*, *CO1*, *CO2*, and *LUX* have previously been described (Yan *et al.*, 2003, 2004, 2006; Turner *et al.*, 2005; Faure *et al.*, 2007, 2012; Kikuchi *et al.*, 2009; Greenup *et al.*, 2010; Campoli *et al.*, 2012*a*, 2013; Zakhrabekova *et al.*, 2012).

### Statistical analysis

All data presented are mean values ±the standard error of the mean. For all experiments where temperature treatments were compared (Figs 2, 3, 5–8; Supplementary Figs S1; 3–6) differences between mean values at every time point were tested by a Student’s *t* test assuming a two-tailed distribution and equal variance. Where greater than two means were compared, an ANOVA test was conducted prior to the Student’s *t* test.

**Fig. 2.**
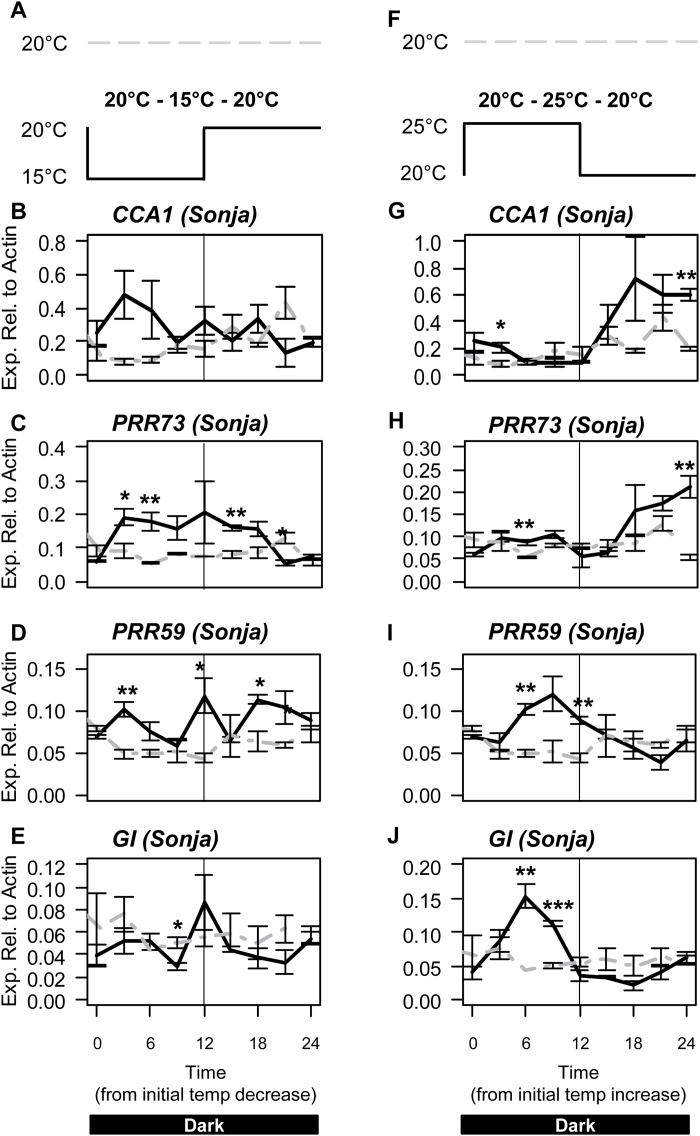
Response of circadian clock genes to temperature change from constant conditions in barley cv. Sonja. Relative expression of circadian clock genes in 5-d-old barley seedlings of cv. Sonja grown at a constant 20 °C (grey dashed line) in constant darkness. At time=0, temperature was either decreased to 15 °C (black line, left panels) or increased to 25 °C (black line, right panels) for 12h and then returned to 20 °C. Values are means of three biological replicates ±standard error. Significant differences are indicated by asterisks (*P<0.05, **P<0.01, ***P<0.001); where no asterisk is present the result is not significant.

## Results

### Temperature entrainment of the barley circadian clock

To determine if the barley circadian clock can be entrained by thermo-cycles, transcript levels of clock genes were examined in barley seedlings cv. Sonja. Seedlings were germinated and grown for 5 d in constant darkness with a daily temperature cycle of approximately 16–25.5 °C (Fig. 1A). Expression of *CCA1*, *TOC1*, *GI*, *PRR73*, *PRR95*, and *PRR59* showed a rhythmic pattern with a period of approximately 24h (Fig. 1B–D).

### Response of clock genes to a transient increase in temperature from a non-oscillating state

To determine which barley clock genes respond rapidly to changes in temperature, the responses of barley clock genes to a transient shift in temperature were examined. Barley seedlings of cv. Sonja were germinated and grown in constant darkness at 20 °C; conditions in which the clock is not oscillating and normal feedback loops are absent (Deng *et al.*, 2015). The temperature was then either increased to 25 °C or decreased to 15 °C for 12h, before being returned to 20 °C (Fig. 2A, F). Gene expression after the temperature shifts was compared with expression in Sonja seedlings grown in constant dark at 20 °C, previously presented in Deng *et al.* (2015). Increased expression of *PRR73* was observed when the temperature decreased from 20 °C to 15 °C and from 25 °C to 20 °C (after the 12h transient increase to 25 °C) (Fig. 2C, H). *CCA1* expression also increased when the temperature was decreased from 25 °C to 20 °C (Fig. 2G). Expression of *PRR59* also increased when the temperature was decreased from 20 °C to 15 °C (Fig. 2D). Transcript levels of *GI*, *PRR73*, and *PRR59* were significantly higher, 6h after the temperature increase from 20 °C to 25 °C and expression began to decline before any other temperature change (Fig. 2H–J). The overall expression pattern of *CCA1*, *GI*, and *PRR59* in response to the temperature increase from 20 °C to 25 °C, appeared to be rhythmic (Supplementary Fig. S2). Expression of *PRR95*, *TOC1*, and *ELF3* were unaffected by changes in temperature (Supplementary Fig. S3).

### Expression profiles of barley clock genes at high versus low ambient temperatures in light/dark cycles

**Fig. 3.**
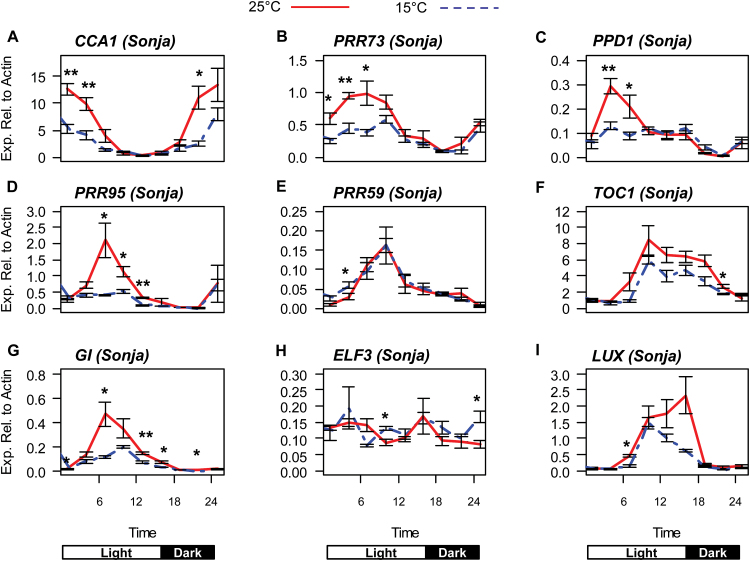
Expression of circadian clock genes in cv. Sonja at 15 °C and 25 °C in long days.Relative expression of circadian clock genes in 5-d-old barley seedlings of cv. Sonja grown at 15 °C (blue dashed line) or 25 °C (red line) in long day (16/8h light/dark) conditions. The labels on the horizontal axis indicate time from lights on. Values are means of three biological replicates ±standard error. Significant differences are indicated by asterisks (*P<0.05, **P<0.01); where no asterisk is present the result is not significant.

To determine the effect of different temperatures on the expression of clock genes in long-day conditions, where the clock is oscillating and temperature impacts growth and development, cv. Sonja seedlings were germinated and grown for 5 d in 16/8h light/dark (long-days) at a constant 15 °C or 25 °C. Under these conditions all clock genes assayed displayed a rhythmic expression profile over the 24h period similar to those described in previous studies (Campoli *et al.*, 2012*b*; Deng *et al.*, 2015). Peak expression of *CCA1*, *PRR73*, *PPD1, PRR95*, and *GI* was significantly higher at 25 °C compared with 15 °C (Fig. 3A–D, G). The *PRR* genes did not all behave the same way; there was little difference between the expression of *PRR59* and *TOC1* at 15 °C compared with 25 °C (Fig. 3E, F). At 25 °C the peak of expression of *LUX* was increased and shifted to the start of the dark period, whereas only minor changes in *ELF3* expression were observed (Fig. 3H, I).

To ensure that differences in the expression of clock genes observed in long days were not an artefact of the temperature treatments chosen, barley seedlings were grown as described above, but at 15, 20, 25, and 30 °C. To maximize any potential differences between the treatments, expression of clock genes was assayed at the time of day when expression was highest (at 25 °C), as previously determined (Fig. 3). Consistent with the previous findings, there was no significant difference in the expression of *TOC1*, *PRR59* or *ELF3* between any of the temperature treatments Supplementary (Fig. S4E, F, H). Although expression of *PRR95* appeared to follow a pattern of increase with increasing temperature, this was not statistically significant (Supplementary Fig. S4D). Expression of *CCA1* increased in a dosage-dependent manner from 15 °C to 25 °C (Supplementary Fig. S4A). *PRR73*, *PPD1*, *GI*, and *LUX* all responded in a similar manner to *CCA1*, although the differences in expression were only statistically significant between the 15 °C and 25 °C temperature treatments (Supplementary Fig. S4B, C, G, I). Expression of all genes, except *LUX*, was lower at 30 °C than at 25 °C and this correlates with a reduction in seedling growth at 30 °C (Supplementary Figs S4; S5). This may indicate that the barley seedlings were experiencing mild heat stress at 30 °C.

To examine which clock genes respond directly to temperature when the clock is oscillating in long-day conditions, barley seedlings were grown at 15 °C and then a subset of seedlings were shifted to 25 °C for 6h. Gene expression in seedlings shifted to 25 °C was compared with expression in seedlings maintained at 15 °C. The temperature shift was timed to coincide approximately with the 6-h period immediately prior to the peak of expression for each gene (Supplementary Fig. S6A). As *PRR59*, *TOC1*, and *ELF3* did not show any significant change in expression in response to temperature (Fig. 3; Supplementary Fig. S4) they were not included in this analysis. Of the clock genes analysed, only *PRR95* showed a significant difference in gene expression between 15 °C and 25 °C within the 6-h treatment (Supplementary Fig. S6B).

### *ELF3* is required for temperature-dependent differences in clock gene expression

**Fig. 4.**
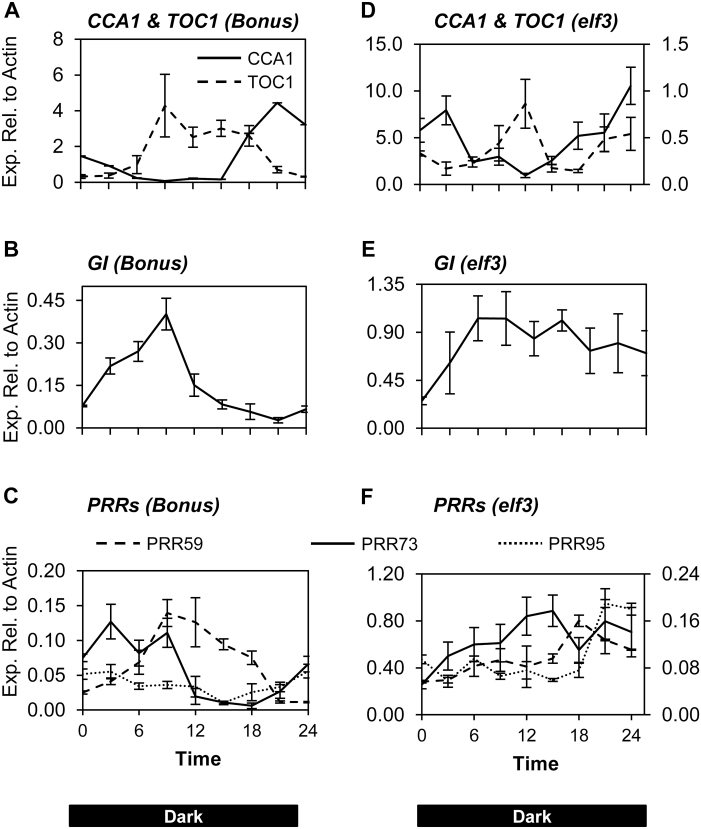
Expression of circadian clock genes in barley cv. Bonus and the *elf3* mutant entrained to temperature cycles in constant darkness. The relative expression of circadian clock genes in 5-d-old barley seedlings, cv. Bonus and *elf3* loss-of-function mutant in Bonus background, grown in constant dark with a daily temperature range of 16–25.5 °C. In *elf3,* the relative expression of *TOC1* and *PRR73* are plotted against the left vertical axis and *CCA1*, *PRR59*, and *PRR95* are plotted against the right vertical axis. Values are means of three biological replicates ±standard error.

The Arabidopsis *ELF3* gene may play a role in integrating temperature cues into the circadian clock (Thines and Harmon, 2010). To examine whether *ELF3* might play such a role in barley, the effect of temperature on the barley *elf3* loss-of-function mutant and the isogenic wild-type parent cv. Bonus were determined. Bonus and *elf3* were germinated and grown in constant darkness with a daily temperature range of approximately 16–25.5 °C (Fig. 1A). Gene expression in cv. Bonus was rhythmic and similar to that observed for cv. Sonja (Fig. 4A–C). In the *elf3* mutant, rhythmic gene expression of *CCA1* and *TOC1* was observed, but the rhythmic expression of *GI*, *PRR73*, *PRR95*, and *PRR59* was severely dampened compared with cv. Bonus (Fig. 4D–F). Expression levels of *CCA1*, *TOC1, GI*, and *PRR73* were increased in the *elf3* loss-of-function mutant compared with cv. Bonus (Fig. 4D–F).

**Fig. 5.**
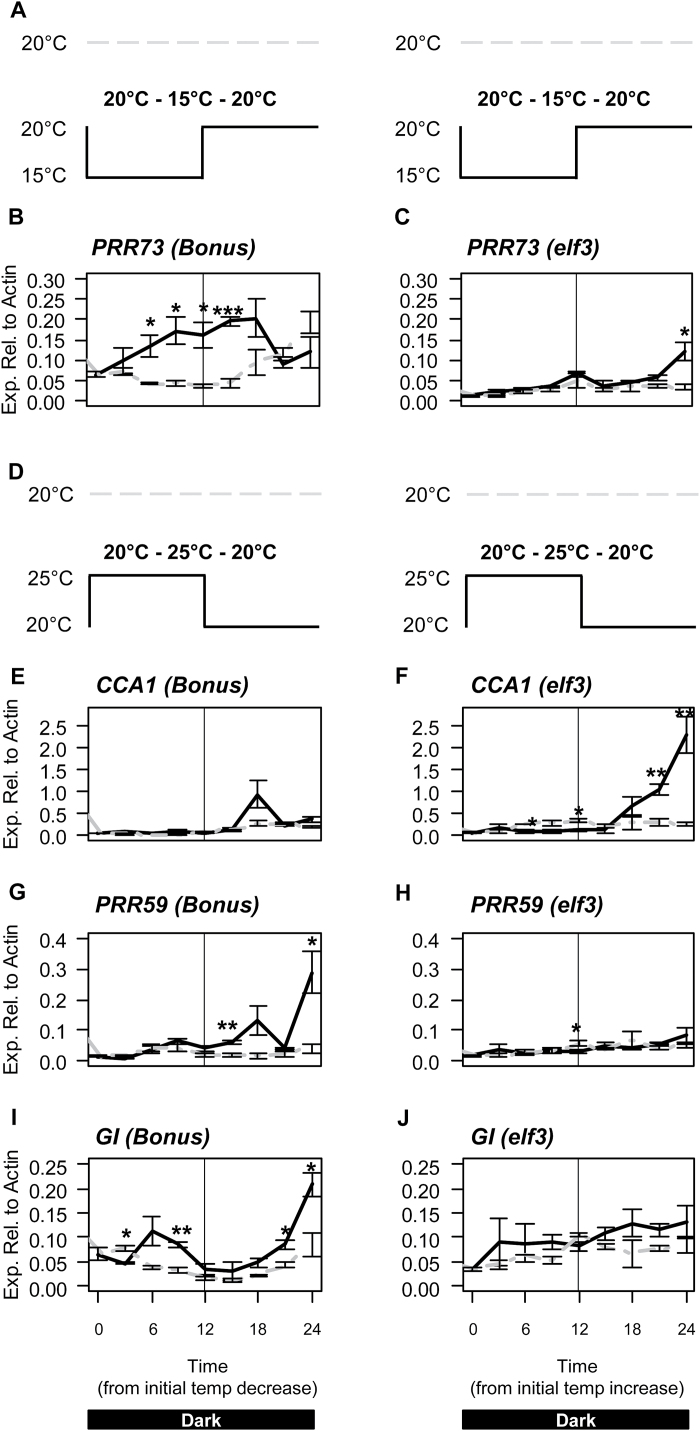
Response of *PRR73*, *CCA1, PRR59*, and *GI* to temperature change from constant conditions in cv. Bonus and *elf3* mutant. The relative expression of *PRR73*, *CCA1, PRR59*, and *GI* in 5-d-old cv. Bonus and *elf3* mutant seedlings grown at a constant 20 °C (grey dashed line) in constant darkness. (**A–C**) At time=0, the temperature was decreased to 15 °C (black line) for 12h and then returned to 20 °C. **(D–J)** At time=0, the temperature was increased to 25 °C (black line) for 12h and then returned to 20 °C. Values are means of three biological replicates ±standard error. Significant differences are indicated by asterisks (*P<0.05, **P<0.01); where no asterisk is present the result is not significant.

In cv. Sonja, expression of *CCA1*, *GI*, *PRR59*, and *PRR73* responded rapidly to a change in temperature from constant conditions (Fig. 2). To determine if these temperature-dependent changes require a functional *ELF3* gene, seedlings of the *elf3* loss-of-function mutant and the isogenic wild-type parent cv. Bonus were germinated and grown for 5 d in constant darkness at 20 °C. The temperature was then either increased to 25 °C or decreased to 15 °C for 12h, before being returned to 20 °C (Fig. 5A, D). In cv. Bonus, *PRR73* expression increased in response to a decrease in temperature from 20 °C to 15 °C similar to that observed in cv. Sonja (Fig. 5B). In the *elf3* mutant, no statistically significant changes in transcript levels were observed for *PRR73* in response to the temperature decrease (Fig. 5C). In cv. Bonus, *GI* expression increased in response to the temperature increase from 20 °C to 25 °C similar to that observed in cv. Sonja, but no significant difference in expression of *GI* was detected in the *elf3* background (Fig. 5I, J). In cv. Bonus and *elf3*, *CCA1* expression increased after the temperature was decreased from 25 °C to 20 °C similar to that observed in cv. Sonja, although the response in cv. Bonus was not as strong as in *elf3* or cv. Sonja (Fig. 5E, F). The response of *PRR59* to the temperature increase from 20 °C to 25 °C observed in cv. Sonja was not present in cv. Bonus, indicating that the genotypic differences between Sonja and Bonus may affect *PRR59* expression (Fig. 5G).

**Fig. 6.**
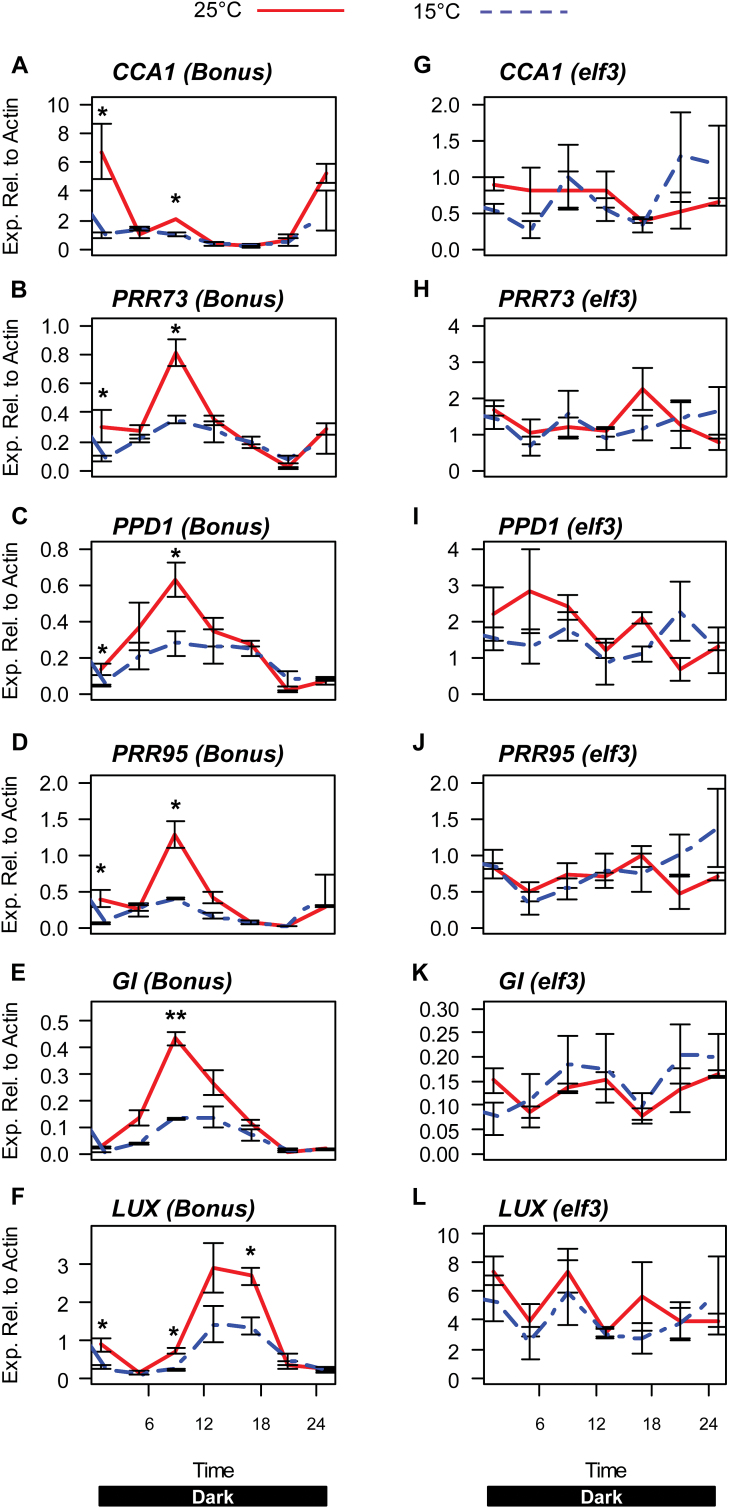
Expression of circadian clock genes at 15 °C and 25 °C in barley cv. Bonus and the *elf3* mutant entrained in long-day conditions. The relative expression of circadian clock genes in barley seedlings cv. Bonus and the *elf3* mutant grown at 15 °C (blue dashed line) or 25 °C (red line). Bonus and *elf3* seedlings were germinated and grown for 4 d in long-day conditions (16/8h light/dark) to entrain the barley clock. Seedlings were then shifted to darkness for 24h and gene expression was sampled every 4h over the next 24h period. The labels on the horizontal axis indicate the time from lights on (from preceding entraining conditions). Values are means of at least three biological replicates ±standard error. Significant differences are indicated by asterisks (*P<0.05, **P<0.01); where no asterisk is present the result is not significant.

To determine whether the observed differences in expression of clock genes in normal oscillating conditions at different temperatures are dependent on a functional circadian clock, gene expression was analysed in the *elf3* loss-of-function mutant. Seedlings of the *elf3* loss-of-function mutant and the isogenic wild-type parent cv. Bonus were germinated and grown at a constant 15 °C or 25 °C in 16/8h light/dark for 4 d to allow for entrainment of the barley circadian clock. Bonus and *elf3* seedlings were then shifted to constant dark, where cv. Bonus maintains rhythmic expression of clock genes, but the barley clock is arrhythmic in the *elf3* background (Faure *et al.*, 2012; Deng *et al.*, 2015). The expression of the core clock genes *CCA1*, *PRR73*, *PPD1*, *PRR95*, *GI*, and *LUX* in cv. Bonus, all showed a rhythmic expression pattern with increased expression at 25 °C, consistent with the changes observed in cv. Sonja (Fig. 6A–F). The expression of the same genes in the *elf3* mutant did not show a rhythmic diurnal expression pattern and there were no significant differences in gene expression between the temperature treatments (Fig. 6G–L).

### Temperature affects expression of genes associated with the long-day promotion of flowering

**Fig. 7.**
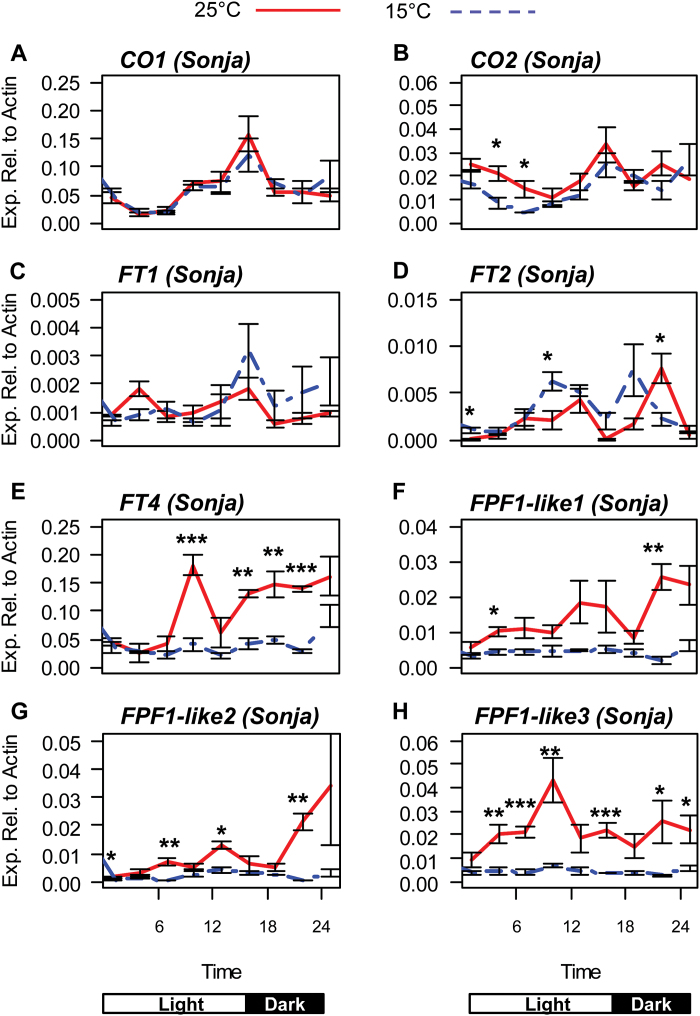
Expression of genes in cv. Sonja associated with the long-day promotion of flowering at 15 °C and 25 °C. The relative expression of flowering time genes in 5-d-old barley seedlings of cv. Sonja grown at 15 °C (blue dashed line) or 25 °C (red line) in long-day (16/8h light/dark) conditions. The labels on the horizontal axis indicate the time from lights on of gene sampling. Values are means of three biological replicates ±standard error. Significant differences are indicated by asterisks (*P<0.05, **P<0.01, ***P<0.001); where no asterisk is present the result is not significant.

To establish if the changes in expression of core clock genes observed at 25 °C correlates with the changes in expression of genes known to promote flowering, the expression level of genes associated with the long-day promotion of flowering was examined at 3-h time intervals in barley seedlings cv. Sonja grown in long-days at constant 25 °C or 15°C. There was no difference in the expression of *CONSTANS 1* (*CO1*) at 25 °C compared with 15 °C (Fig. 7A). A small but statistically significant increase in *CO2* expression was detected at 25 °C early in the light period (Fig. 7B). Barley has five members of the *FLOWERING LOCUS T* (*FT*) gene family (*FT1*, *2*, *3*, *4*, and *5*) and one *TERMINAL FLOWERING 1* (*TFL1*) homologue. *FT3* is deleted in cv. Sonja and expression of *FT5* and *TFL1* could not be detected. Expression of *FT1* and *FT2* was detectable. There was no significant difference in expression of *FT1* at 15 °C compared with 25 °C but small differences in expression of *FT2* were observed (Fig. 7C, D). Expression of *FT4* was significantly increased at 25 °C, especially in the dark (Fig. 7E). Next, the expression of three *FLOWERING PROMOTING FACTOR 1-like* (*FPF1-like*) genes that respond to high temperature were determined (Hemming *et al.*, 2012)*. FPF1-like 1, 2*, and *3* all had significantly higher expression at 25 °C (Fig. 7F–H).

**Fig. 8.**
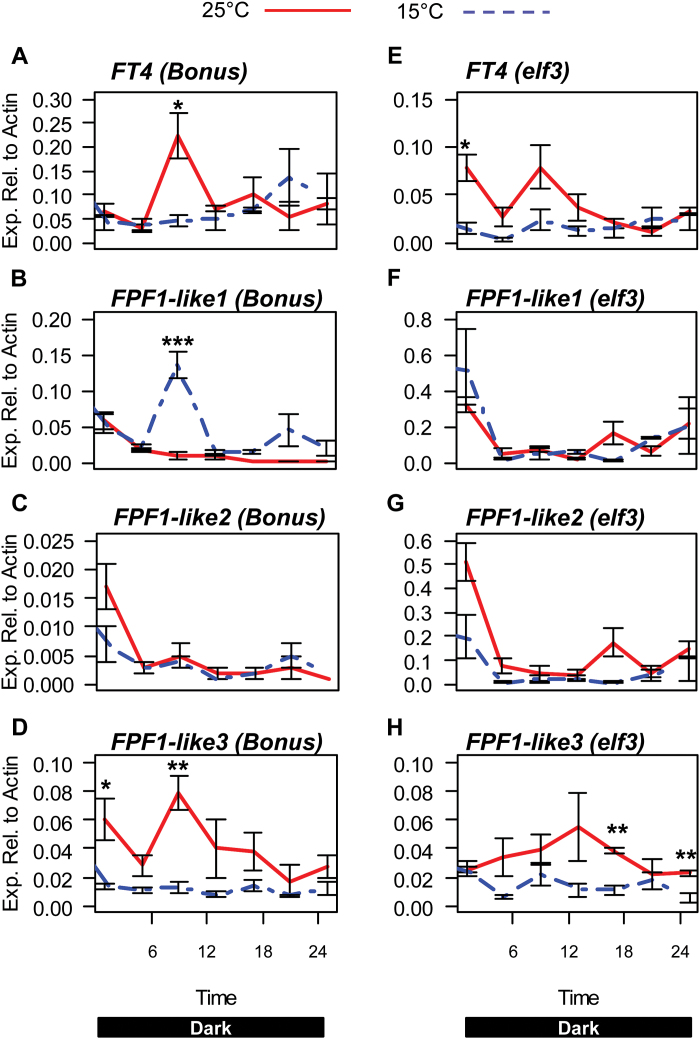
Expression of genes associated with the long-day. promotion of flowering at 15 °C and 25 °C in barley cv. Bonus and *elf3* mutants. The relative expression of circadian clock genes in barley seedlings cv. Bonus and *elf3* mutant grown at 15 °C (blue dashed line) or 25 °C (red line). Bonus and *elf3* seedlings were germinated and grown for 4 d in long-day conditions (16/8h light/dark) to entrain the barley clock. Seedlings were then moved into darkness for 24h and gene expression was sampled every 4h over the next 24h period. The labels on the horizontal axis indicate the time from lights on (from preceding entraining conditions). Values are means of at least three biological replicates ±standard error. Significant differences are indicated by asterisks (*P<0.05, **P<0.01, ***P<0.001); where no asterisk is present the result is not significant.

To determine if any of the observed changes in flowering time genes were under circadian control, the expression of *FT4*, *FPF1-like 1*, *2*, and *3* was examined in the *elf3* loss-of-function mutant in conditions where the clock is arrhythmic. As described previously, the isogenic wild-type parent cv. Bonus and *elf3* mutant seedlings were entrained in long-day conditions at a constant 15 °C or at 25 °C and then transferred to constant darkness, where *elf3* is arrhythmic. An initial increase in *FT4* expression at 25 °C was observed in cv. Bonus, similar to the initial peak of expression observed in cv. Sonja (Fig. 8A). In the *elf3* background there was also a significant increase in *FT4* expression, although peak expression occurred 6h earlier than observed in cv. Bonus (Fig. 8E). Expression of *FPF1-like 3* was also increased in both cv. Bonus and the *elf3* mutant (Fig. 8D, H).The increased expression of *FPF1-like 1* and *2* previously seen in cv. Sonja at 25 °C was not observed in either cv. Bonus or the *elf3* mutant (Fig. 8B, C, F, G).

## Discussion

### The barley circadian clock can be entrained by temperature and maintain rhythmic gene expression at different temperatures

A key feature of the plant circadian clock is the capacity to be entrained to thermo-cycles (‘temperature entrainment’). Here we show that when barley seedlings are germinated and grown in constant darkness with oscillating temperature, core clock genes display robust rhythms with a period of approximately 24h (Fig. 1). These data show that, as in other plant species, the barley clock is competent to be entrained by thermo-cycles. Interestingly, a 12h temperature shift was not sufficient to initiate robust rhythms in all clock genes, although the differences in the peaks and troughs of *CCA1*, *PRR59*, and *GI* expression, detected after the temperature was increased from 20 °C to 25 °C, appear to resemble normal clock rhythms (Fig. 2; Supplementary Fig. S2). This is different from the response to light where one light period was sufficient to initiate clock rhythms in all clock genes (Deng *et al.*, 2015).

Another key feature of the plant circadian clock is the capacity to maintain the period of rhythmic gene expression across a range of temperatures. In this study, we show that there was no difference in the timing of daily peaks and troughs of clock gene expression in barley seedlings, grown for 5 d, in long days, at either 15 °C or 25 °C (Fig. 3). This demonstrates that the barley clock appears to be able to maintain the same period of expression of clock genes at different temperatures.

Despite evidence implicating the circadian clock in the control of flowering time in cereals, the way in which temperature affects the cereal clock and its involvement in temperature-related growth and development is not well understood. In this study, we demonstrate that four clock genes, *GI, CCA1, PRR59*, and *PRR73*, show rapid and significant changes in expression levels from a non-oscillating state when exposed to changes in temperature (Fig. 2). *GI* and *CCA1* have previously been shown to be important for light entrainment of the barley circadian clock (Deng *et al.*, 2015). Expression of *GI* increases rapidly in response to a transition from dark to light (Deng *et al.*, 2015) and in response to a transition from a cool to a warm temperature (Fig. 2), both of which are indicative of a dawn cue. Similarly, *CCA1* expression increases rapidly in response to a transition from light to dark (Deng *et al.*, 2015) and to a transition from warm to cool (Fig. 2), both of which are indicative of a dusk cue. The expression of *GI*, *CCA1*, and *PRR59* also appeared to become rhythmic after the temperature was increased from 20 °C to 25 °C (Fig. 2; Supplementary Fig. S2). Given the common response of *GI* and *CCA1* to both temperature and light cues, these genes may play a role in setting daily circadian rhythms in barley.

In long-day conditions, expression levels of *CCA1*, *PRR73*, *PPD1*, *PRR95*, *GI*, and *LUX* were all increased at 25 °C compared with 15 °C, but rhythmic gene expression was maintained (Fig. 3). To determine which genes might be important for this transcriptional response to temperature, barley seedlings were grown at 15 °C and the temperature was increased to 25 °C for 6h. Of all the clock genes analysed, only *PRR95* showed an increase in expression in response to a temperature increase in long-day conditions (Supplementary Fig. S6). *PRR95* has also been shown to respond strongly to light (Deng *et al.*, 2015), suggesting that it may be important for integrating temperature and light cues into the barley circadian clock.

It is important to note that the clock genes that respond to a change in temperature from constant conditions are different to those from oscillating conditions. In constant conditions, *CCA1*, *PRR59*, *PRR73*, and *GI* all responded rapidly to changes in temperature (Fig. 2). In long-day oscillating conditions only *PRR95* responded to a change in temperature (Supplementary Fig. S6). The response of clock genes to changes in temperature may be dependent on the state of the clock or light. Given the limited response of clock genes to temperature changes in oscillating conditions, constant (non-oscillating) conditions may be best to analyse the affects of temperature on the barley clock. A common factor across all temperature experiments was the responsiveness of *CCA1*, *PRR59*, *PRR73*, *PRR95*, and *GI* to different temperature treatments. This implies that *CCA1*, *GI*, and the *PRR* genes have an important role in the temperature responses of the barley clock.

### Genes associated with the long-day promotion of flowering respond to temperature in a clock-independent manner

In cv. Sonja, expression of *FT4*, *FPF1-like 1*, *2*, and *3* was increased at 25 °C compared with 15 °C (Fig. 7). In cv. Bonus, the significant increase in expression of *FPF1-like 1* and *2* was not detected (Fig. 8). This may be due to differences in the genetic backgrounds of these genotypes. Similar to cv. Sonja, increased expression of *FT4* and *FPF1-like 3* was detected at higher temperature in cv. Bonus and in *elf3* (Fig. 8). under these conditions, normal rhythmic expression and the temperature response of clock genes is lost in the *elf3* mutant (Figs 6, 8). This suggests that the up-regulation of *FT4* and *FPF1-like 3,* while potentially important in temperature-dependent flowering, may be independent of the circadian clock.

Expression of *PPD1,* the major determinant of photoperiod-dependent flowering in barley, is increased at 25 °C, although no increase in expression was observed in the downstream floral promoting gene *FT1* (Fig. 7). This is similar to previous work that showed no difference in the transcript levels of *FT1* in early-flowering barley plants grown at 25 °C compared with those grown at 15 °C (Hemming *et al.*, 2012). The strong up-regulation of *PPD1* at 25 °C may be important for accelerating flowering at warm temperatures, but potential downstream targets are still to be identified.

### The barley *ELF3* gene plays a role in the response of clock genes to warm temperature cues

The barley circadian clock was able to be entrained by temperature in cvs Sonja and Bonus but not in the *elf3* loss-of-function mutant, indicating that *ELF3* is important for temperature entrainment (Figs 1, 4). *ELF3*’s role in temperature entrainment appears to be conserved, as the Arabidopsis *ELF3* gene has also been shown to be important for temperature entrainment (Thines and Harmon, 2010). In cv. Sonja, temperature changes resulted in altered expression of *GI* and the *PRR* genes. In the barley *elf3* loss-of-function mutant, in which functioning of the circadian clock is compromised, changes in expression of *GI*, *PRR59*, and *PRR73,* observed in response to a rapid temperature shift from constant conditions were lost (Fig. 5). The increased expression of *GI*, *PRR73*, and *PRR95*, at 25 °C in normal oscillating conditions were also lost in the *elf3* mutant (Fig. 6). This indicates a potential role for *ELF3* in mediating these transcriptional responses to temperature. As shown previously, and in this study, expression of *PRR* genes and *GI* is constitutively increased in the barley *elf3* mutant, suggesting that these genes are negatively regulated by *ELF3* (Faure *et al.*, 2012; Zakhrabekova *et al.*, 2012; Deng *et al.*, 2015). The increase in expression of *GI, LUX*, and the *PRR* genes at 25 °C observed in this study, may be due to the removal of *ELF3*-mediated repression. Interestingly, no changes in expression of *ELF3* were observed in response to changes in temperature indicating that *ELF3* may be post-transcriptionally regulated by temperature. In Arabidopsis, ELF3 together with ELF4 and LUX form the evening protein complex that represses *GI* and the *PRR* genes (Dixon *et al.*, 2011; Helfer *et al.*, 2011; Nusinow *et al.*, 2011; Mizuno *et al.*, 2014). Warm temperatures have also been proposed negatively to regulate the evening complex in *Arabidopsis* (Mizuno *et al.*, 2014). A similar mechanism may exist in barley, although this has yet to be confirmed.

Warm temperatures are known to alter the vegetative and reproductive development of barley (Hemming *et al.*, 2012). In addition, *ELF3* has been shown to regulate photoperiod-dependent flowering and gibberellic acid (GA) synthesis, altering growth and development (Turner *et al.*, 2005; Faure *et al.*, 2012; Hemming *et al.*, 2012; Zakhrabekova *et al.*, 2012; Campoli *et al.*, 2013;Boden *et al.*, 2014). The *elf3*-dependent changes in the expression of barley clock genes identified in this study may be a potential mechanism controlling warm temperature-dependent growth and development in barley.

## Conclusion

In this study we examined the relationship between temperature and expression of core genes in the barley circadian clock. We show that the barley circadian clock can integrate temperature cues to entrain, and compensate for, changes in temperature. In barley, the expression levels of the core clock genes *CCA1*, *GI*, *PRR59*, *PRR73*, *PRR95*, *PPD1*, and *LUX* change in response to changes in temperature. These data show that some clock genes are temperature responsive at the transcript level in barley. In barley, the responses of the circadian clock genes and floral promoting genes analysed in this study do not appear to be linked. If a temperature- and clock-dependent flowering mechanism exists in cereals, further investigation into the significance of the increase in *PPD1* expression at high temperature may prove a good starting point for identifying such a mechanism.

Yields of the world’s major cereal crops are predicted to fall as global temperatures rise; these data will underpin future studies on cereal development, flowering, and grain yield under increasing temperature.

## Supplementary data

**Figure S1.** Comparison of *ACTIN* and *GAPDH* reference genes.

**Figure S2.** Expression of *CCA1*, *GI*, and *PRR59* after a 12h temperature increase from constant conditions.

**Figure S3.** Response of *PRR95*, *TOC1*, and *ELF3* to a 12h temperature change in cv. Sonja.

**Figure S4.** Expression of circadian clock genes in cv. Sonja at 15, 20, 25, and 30 °C.

**Figure S5.** Barley seedling growth at different temperatures.

**Figure S6.** Expression of circadian clock genes in cv. Sonja after a 6-h temperature pulse.

**Table 1.** qRT-PCR primers used in this study.

Supplementary Data
