# Supplementary material for: Barley (Hordeum vulgare) circadian clock genes can respond rapidly to temperature in an EARLY FLOWERING 3-dependent manner
Source: J Exp Bot. 2016 Aug 31;67(18):5517–28. doi: 10.1093/jxb/erw317 (PMC5049398; doi:10.1093/jxb/erw317)
Supplement: Supplementary Data [file supp_67_18_5517__index.html]

Barley (Hordeum vulgare) circadian clock genes can respond rapidly to temperature in an EARLY FLOWERING 3-dependent manner — Barley (Hordeum vulgare) circadian clock genes can respond rapidly to temperature in an EARLY FLOWERING 3-dependent manner — Supplementary Data 

# Barley (*Hordeum vulgare*) circadian clock genes can respond rapidly to temperature in an *EARLY FLOWERING 3*-dependent manner

## Supplementary Data

Data files

- Supplementary\_figures\_S1\_S6.pdf - Supplementary Data
- Supplementary\_table\_1.docx - Supplementary Data
