# Supplementary material for: Barley (Hordeum vulgare) circadian clock genes can respond rapidly to temperature in an EARLY FLOWERING 3-dependent manner
Source: J Exp Bot. 2016 Aug 31;67(18):5517–28. doi: 10.1093/jxb/erw317 (PMC5049398; doi:10.1093/jxb/erw317)
Supplement: Supplementary Data [file supp_erw317_Supplementary_figures_S1_S6.pdf]

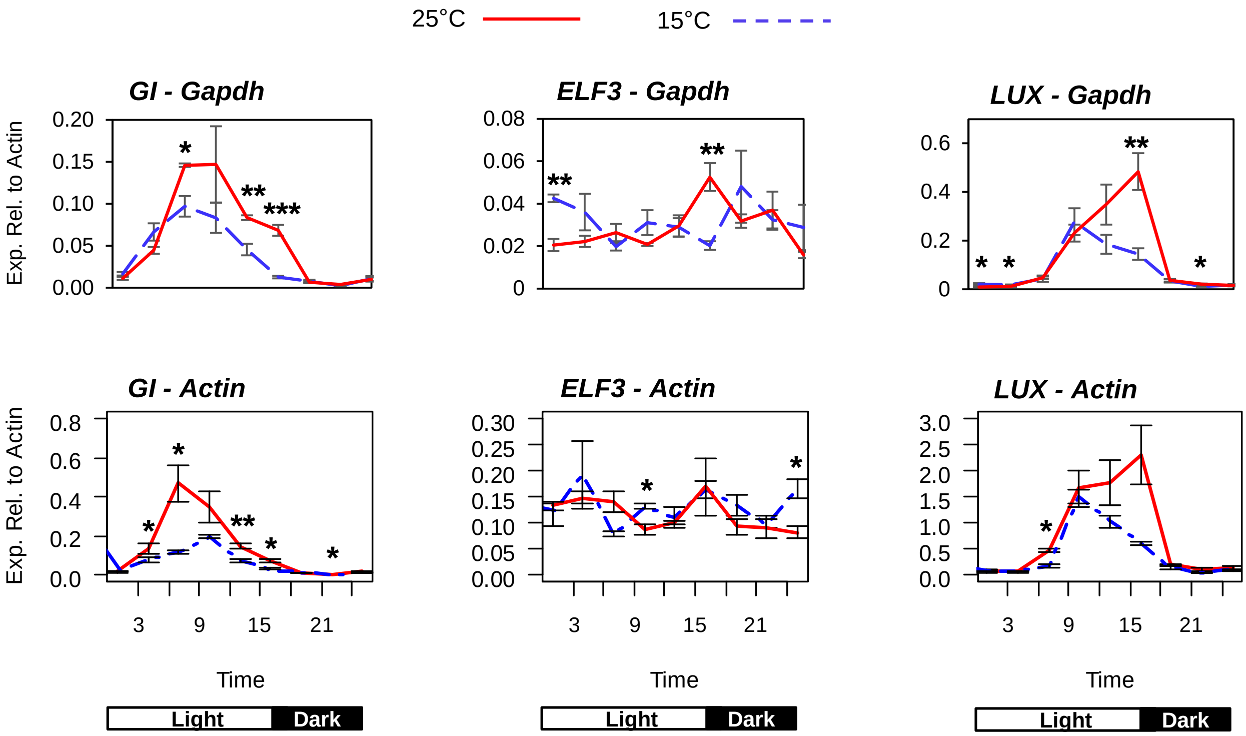

Figure S1. Comparison of *ACTIN* and *GAPDH* reference genes

Relative expression of *GI*, *ELF3* and *LUX* comparing *ACTIN* and *GAPDH* reference genes in barley seedlings cv. Sonja. Barleys were grown at a constant 15°C (blue lines) and 25°C (red lines) in 16h light:8h dark long days. Values are means of three biological replicates ± standard error. Significant differences are indicated by asterisks (**p* < *0.05*, ***p* < *0.01*, ****p* < *0.001*) where no asterisk is present the result is not significant.

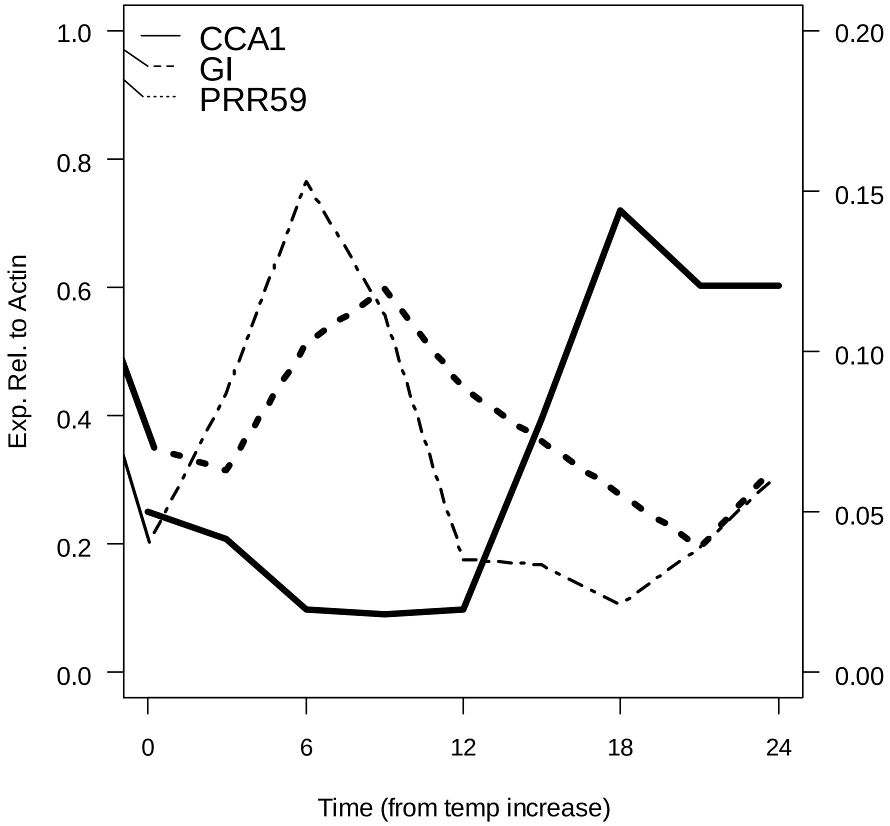

Figure S2 Expression of *CCA1*, *GI* and *PRR59* after 12 hour increase in temperature from 20^{0}C to 25^{0}C from constant conditions

Combined plot of relative expression of *CCA1*, *GI* and *PRR59* after 12 hour temperature increase from 20^{0}C to 25^{0}C from Figure 2. Relative expression of *CCA1* is plotted against the left y-axis and *GI* and *PRR59* are plotted against the right y-axis.

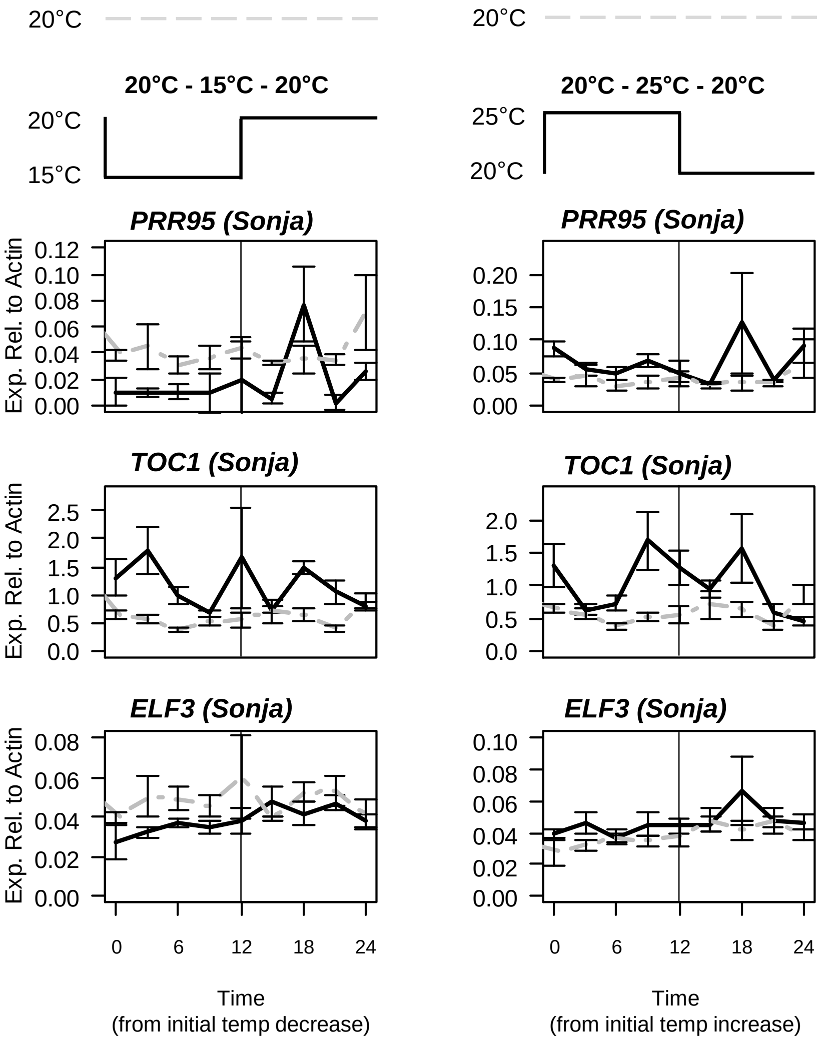

Figure S3 Response of circadian clock genes *PRR95*, *TOC1* and *ELF3* to an initial 12 hour temperature change.

Relative expression of circadian clock genes in five day old barley seedlings cv. Sonja grown at a constant 20^{0}C (grey dashed line) in constant dark. At time = 0, temperature was either decreased to 15^{0}C (black line, left panels) or increased to 25^{0}C (black line, right panels) for twelve hours and then returned to 20^{0}C. Values are means of three biological replicates ± standard error. Significant differences are indicated by asterisks (***p < 0.01*) where no asterisk is present the result is not significant.

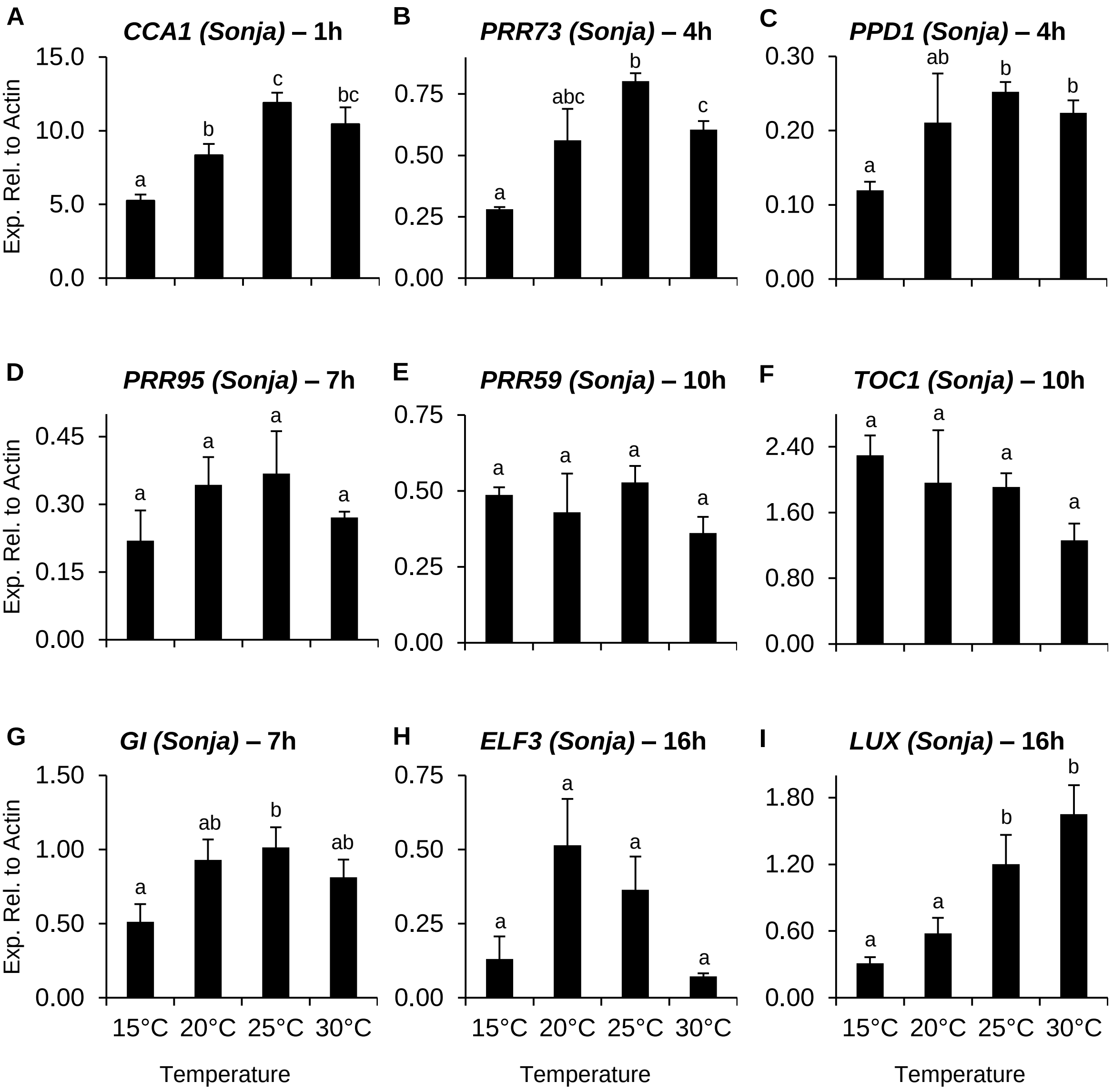

Figure S4 Expression of circadian clock genes in cv. Sonja at 15^{0}C, 20^{0}C, 25^{0}C and 30^{0}C.

Relative expression of circadian clock genes in five day old barley seedlings cv. Sonja grown at 15^{0}C, 20^{0}C, 25^{0}C or 30^{0}C in long day (16 hours light:8 hours dark) conditions. Gene expression was measured at the point of peak expression as determined in Figure 3. Time refers to the number of hours from lights on of gene sampling. Values are means of three biological replicates ± standard error. Pairs of means with the same lowercase character are not significantly different from each other ($p < 0.05$).

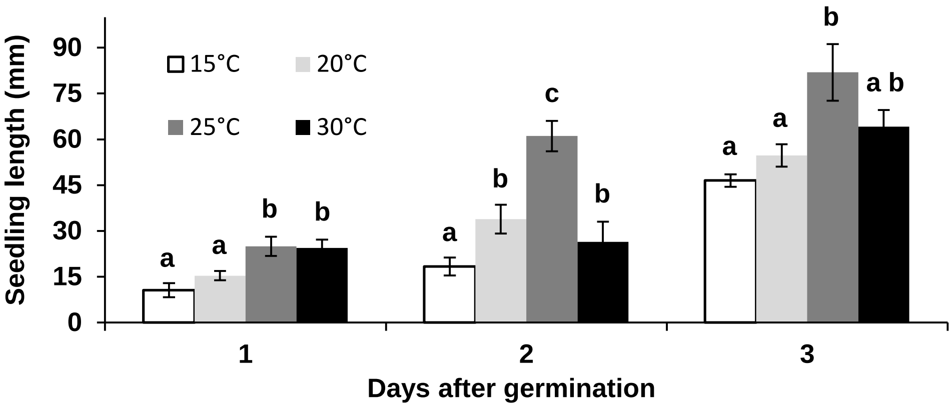

Figure S5 Barley seedling growth at different temperatures.

Seedling length cv. Sonja grown at a constant 15^{0}C, 20^{0}C, 25^{0}C or 30^{0}C. Values are means of at least seven biological replicates ± standard error. Pairs of means with the same lowercase character are not significantly different from each other ($p < 0.05$).

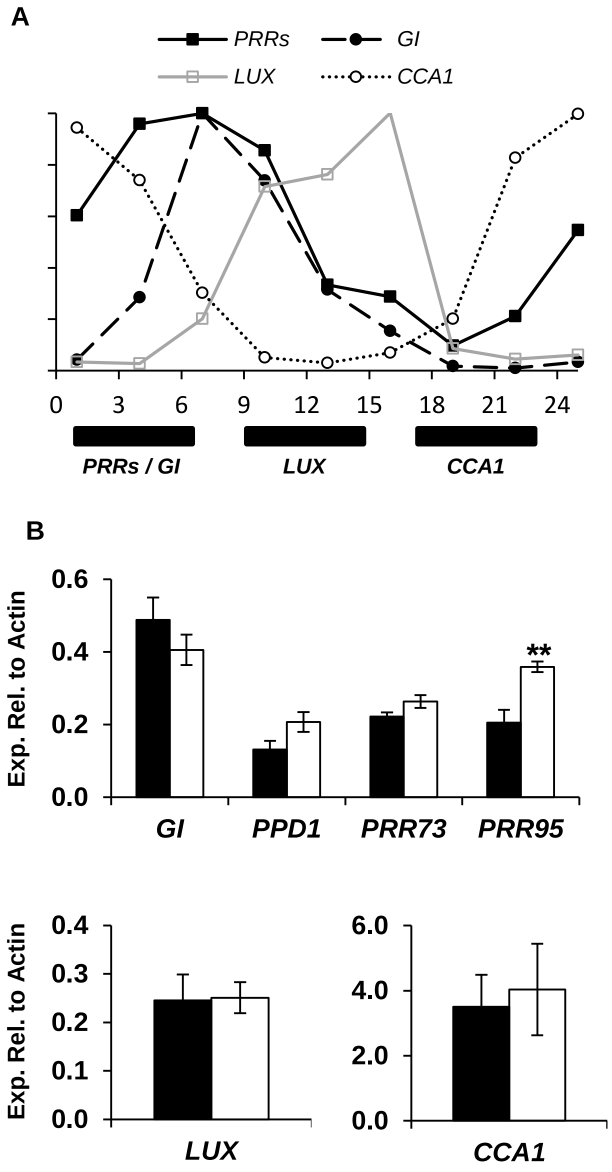

Figure S6 Expression of circadian clock genes in cv. Sonja after a six hour temperature pulse.

Relative expression of circadian clock genes in five day old barley seedlings cv. Sonja grown at 15^{0}C in long day (16 hours light:8 hours dark) conditions and then maintained at 15°C or exposed to a six hour temperature increase to 25^{0}C. **A.** Daily expression patterns of *CCA1*, *GI*, *LUX* and *PRR* genes. Six hour temperature pulses (black bars) were timed to finish at approximately the point of peak gene expression. **B.** Gene expression after six hours at 15^{0}C (black bars) and after six hours at 25^{0}C (open bars). Values are means of three biological replicates ± standard error. Significant differences are indicated by asterisks (**$p < 0.01$) where no asterisk is present the result is not significant.
