# Supplementary material for: Barley (Hordeum vulgare) circadian clock genes can respond rapidly to temperature in an EARLY FLOWERING 3-dependent manner
Source: J Exp Bot. 2016 Aug 31;67(18):5517–28. doi: 10.1093/jxb/erw317 (PMC5049398; doi:10.1093/jxb/erw317)
Supplement: Supplementary Data [file supp_erw317_Supplementary_table_1.docx]

Supplementary Table 1 qRT-PCR primers used in this study

| Gene | Direction | Sequence 5’ – 3’ | Reference |
| --- | --- | --- | --- |
| *CO2* | F | AGTGGACTCTTGGCTCCTCA | Campoli *et al.* 2012 |
|  | R | CATGCTGCTGTTCTTGCATT |  |
| *FT1* | F | ATGAGGACCTTCTACACGCT | Hemming *et al.* 2012 |
|  | R | GGCTCTCGTACCACATCACC |  |
| *FT2* | F | GGATCCGGATGCTCCAAG | Faure *et al.* 2007 |
|  | R | CATCACATCCTTCTCCCG |  |
| *FT4* | F | GATGTCCCAGCATCAACA | Faure *et al.* 2007 |
|  | R | CAGCTGCTGGTACAGAAC |  |
| *FPF1-like 1* | F | GTCAAGAACCGCGACCACTT | Greenup *et al.* 2010 |
|  | R | CACATGCATAATCGCACACG |  |
| *FPF1-like 2* | F | CGTCGACCTCATCTCCCTTC | Greenup *et al.* 2010 |
|  | R | AGTTGATTTGGGGCAGCTTG |  |
| *FPF1-like 3* | F | CGCTACTACGAGAACCGAGACATC | Hemming *et al.* 2012 |
|  | R | CCTTGAAGTGGTGCCTGTTCTGG |  |
| *VRN1* | F | GGAAACTGAAGGCGAAGGTTGA | Greenup *et al.* 2010 |
|  | R | TGGTTCTTCCTGGCTCTGATATGTT |  |
